# redbiom: a Rapid Sample Discovery and Feature Characterization System

**DOI:** 10.1128/mSystems.00215-19

**Published:** 2019-06-25

**Authors:** Daniel McDonald, Benjamin Kaehler, Antonio Gonzalez, Jeff DeReus, Gail Ackermann, Clarisse Marotz, Gavin Huttley, Rob Knight

**Affiliations:** a Department of Pediatrics, University of California San Diego, La Jolla, California, USA; b School of Science, University of New South Wales, Canberra, Australia; c Research School of Biology, Australian National University, Canberra, Australia; d Department of Computer Science and Engineering, University of California San Diego, La Jolla, California, USA; e Center for Microbiome Innovation, University of California San Diego, La Jolla, California, USA; Institute of Soil Science, Chinese Academy of Sciences

**Keywords:** database, meta-analysis, microbiome

## Abstract

Meta-analyses at the whole-community level have been important in microbiome studies, revealing profound features that structure Earth’s microbial communities, such as the unique differentiation of microbes from the mammalian gut relative to free-living microbial communities, the separation of microbiomes in saline and nonsaline environments, and the role of pH in driving soil microbial compositions. However, our ability to identify the specific features of a microbiome that differentiate these community-level patterns have lagged behind, especially as ever-cheaper DNA sequencing has yielded increasingly large data sets. One critical gap is the ability to search for samples that contain specific features (for example, sub-operational taxonomic units [sOTUs] identified by high-resolution statistical methods for removing amplicon sequencing errors). Here we introduce redbiom, a microbiome caching layer, which allows users to rapidly query samples that contain a given feature, retrieve sample data and metadata, and search for samples that match specified metadata values or ranges (e.g., all samples with a pH of >7), implemented using an in-memory NoSQL database called Redis. By default, redbiom allows public anonymous sample access for over 100,000 publicly available samples in the Qiita database. At over 100,000 samples, the caching server requires only 35 GB of resident memory. We highlight how redbiom enables a new type of characterization of microbiome samples and provide tutorials for using redbiom with QIIME 2. redbiom is open source under the BSD license, hosted on GitHub, and can be deployed independently of Qiita to enable search of proprietary or clinically restricted microbiome databases.

**IMPORTANCE** Although analyses that combine many microbiomes at the whole-community level have become routine, searching rapidly for microbiomes that contain a particular sequence has remained difficult. The software we present here, redbiom, dramatically accelerates this process, allowing samples that contain microbiome features to be rapidly identified. This is especially useful when taxonomic annotation is limited, allowing users to identify environments in which unannotated microbes of interest were previously observed. This approach also allows environmental or clinical factors that correlate with specific features, or vice versa, to be identified rapidly, even at a scale of billions of sequences in hundreds of thousands of samples. The software is integrated with existing analysis tools to enable fast, large-scale microbiome searches and discovery of new microbiome relationships.

## Abstract (precis)

Although analyses that combine many microbiomes at the whole-community level have become routine, searching rapidly for microbiomes that contain a particular sequence has remained difficult. The software we present here, redbiom, dramatically accelerates this process, allowing samples that contain microbiome features to be rapidly identified. This is especially useful when taxonomic annotation is limited, allowing users to identify environments in which unannotated microbes of interest were previously observed. This approach also allows environmental or clinical factors that correlate with specific features, or vice versa, to be identified rapidly, even at a scale of billions of sequences in hundreds of thousands of samples. The software is integrated with existing analysis tools to enable fast, large-scale microbiome searches and discovery of new microbiome relationships.

## OBSERVATION

Data reuse has posed a significant challenge in the microbiome field, especially because of technical variation among studies (1). Analyses at the whole-community level, typically using principal-coordinate analysis (PCoA) or similar dimensionality reduction techniques, have nevertheless revealed many large-scale patterns relating microbiomes to one another (2–4), especially when standardized techniques are used either within one study or across many studies in a consortium effort using common protocols (5). In particular, resources such as Qiita (6) were developed to facilitate reuse of data and now house amplicon data from hundreds of thousands of microbiomes with associated metadata (per-sample, per-individual, and/or per-site information related to each sample) in the standardized MIxS format introduced by the Genomic Standards Consortium (7).

There is a need to search for samples that contain particular microbial taxa and for taxa that explain differences among samples. These tasks are especially important for revealing which specific microbes are associated with particular environmental or clinical metadata. Performing the search directly at the sequence level is possible, but typically incurs substantial computational effort, especially as improvements in sequencing technology yield ever-larger data sets. To address this need, we developed redbiom, which enables rapid discovery and retrieval of sample data into BIOM tables (8) for immediate integration for meta-analysis.

**FIG 1:**
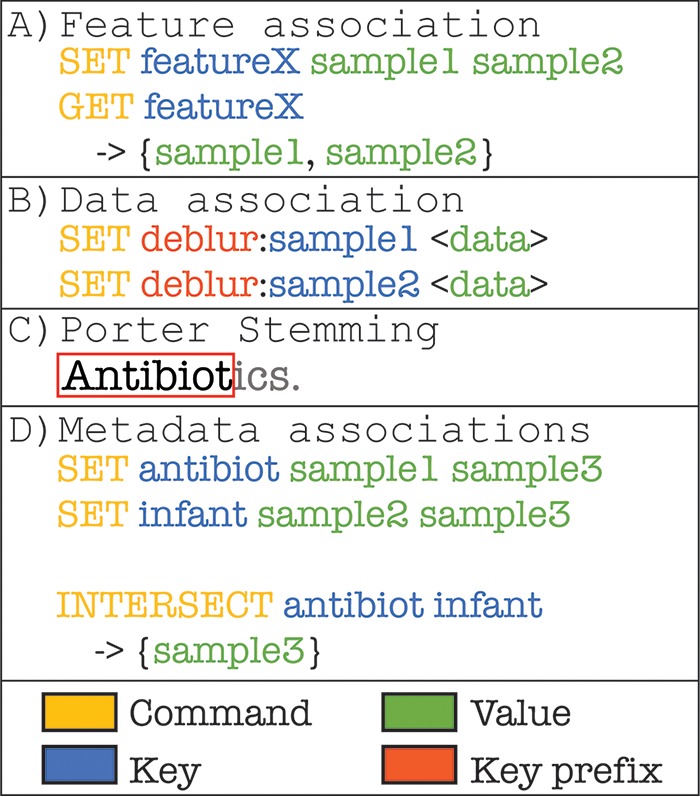
The redbiom data model is a key-value store built on top of Redis. By storing features and sample identifiers as keys, it is possible to rapidly query the resource for information on those entities. Similarly, by indexing the sample metadata, queries can be performed against variables of interest (e.g., pH) in order to identify sample identifiers of interest, which can then be used to extract a feature table for downstream analysis. (A) A “set” command associates a key with a value: in this case, a feature identifier is associated with the samples the feature was observed in. A “get” command can then be issued using the feature identifier as the key to obtain the associated values (i.e., the samples). (B) Feature counts (e.g., a vector from an OTU table) are associated with a composite key that describes the processing context and the sample identifier. The processing context, in this case “deblur,” denotes a bioinformatic procedure applied. For Qiita, the context names also include molecular preparation details. The expectation is the data within a context should be comparable. The sample data themselves are encoded in a sparse vector format with the feature identifiers remapped into unique integers to improve compression and reduce data redundancy. (C) The Porter stem of the word “Antibiotics.” (D) The association of metadata word stems with sample identifiers. Redis natively supports classic set operations, which can be applied to keys to obtain, for example, the intersection of sample identifiers represented by two keys.

Figure 1 outlines the redbiom data model. At its core, redbiom is a structured data model built off Redis, a key-value in memory NoSQL database. Sample data are stored in sparse vectors allowing hundreds of thousands of samples with multiple different processing to be represented in under 40 GB (underlying sequence data total of 45 TB). Identifiers are remapped into a unique integer space to minimize memory utilization and to leverage Redis ziplist optimizations. Data are partitioned by sequencing and bioinformatics protocol to minimize technical biases. These partitions, called “contexts,” allow for identifying samples processed in one way (e.g., Deblur [9]) and obtaining data from another (e.g., closed reference operational taxonomic unit [OTU] picking). Sample and preparation information are indexed efficiently and allow retrieval of a specific variable for a given sample. These variables are additionally indexed by applying Porter stemming (10) to all unique strings such that each stem forms a key that is associated with a set containing the samples where that stem was observed. The combination of indexing strategies allows users to generally search for samples (e.g., all samples with the stem of antibiotics) or to constrain the searches to specific variables and values (e.g., all samples with “soil” in the description field and a pH of <7).

redbiom enables a new paradigm for microbiome analysis and data mining. With indexed exact sequences, it is possible to perform a maximal-precision search of deposited studies to test for replication (as noted in reference 11 [example below]). This is in contrast to manually identifying studies and processing and searching existing raw data or the more frequent strategy of relying on imprecise taxon names mentioned in manuscripts (e.g., hunting for *Clostridium* sp. enrichment in human fecal studies). As redbiom indexes sample metadata and taxonomic information (when available), it also readily allows users to identify samples of interest for comparative purposes: e.g., “How do my samples compare to the Earth Microbiome Project soil samples?” By partitioning technical parameters, it is possible to identify samples in one context and extract from another (e.g., selecting samples with closed reference OTUs based on the presence of specific 16S Deblur sub-operational taxonomic units [sOTUs]).

**FIG 2:**
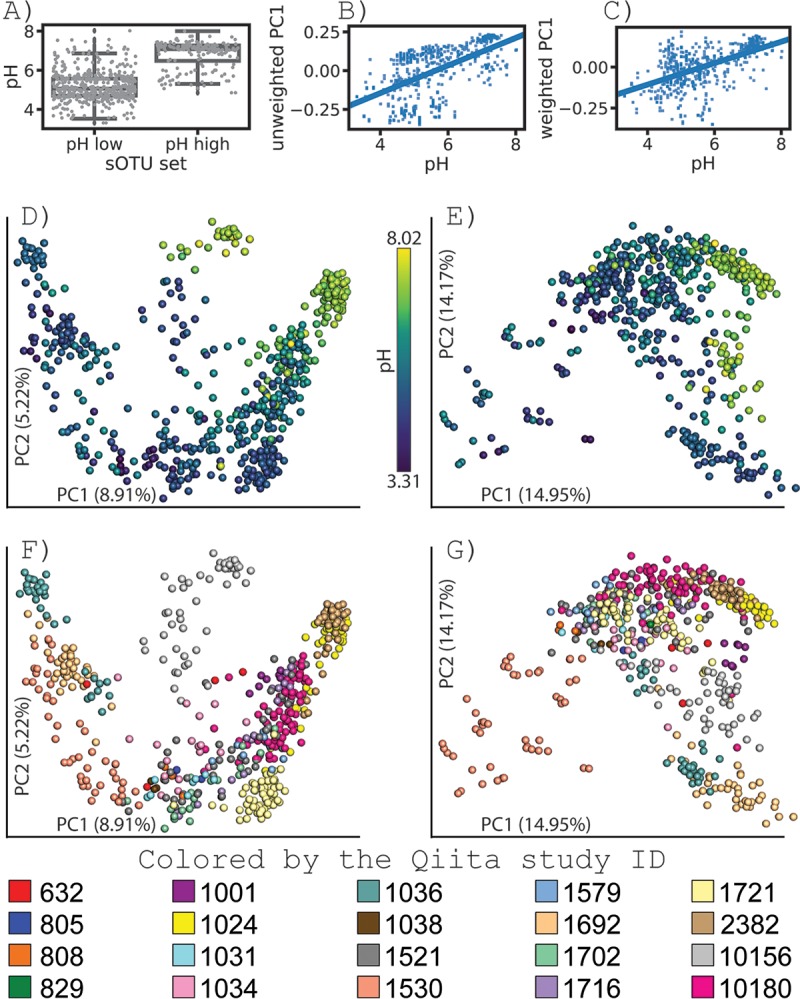
Feature search example. Differential sOTUs from a reanalysis of the study by Ramirez et al. (13) by Morton et al. (unpublished), characterized as associating with a low- or high-pH soil, were obtained. Features were trimmed to 90 nucleotides (nt) to maximize overlap of the Earth Microbiome Project and were searched using redbiom against the Deblur 16S V4 90-nt context with the following sample constraints: “where empo_3=='Soil (non-saline)' and ph > 0.” All samples from Ramirez et al. were removed to create a sample set independent from the observation source: 560 samples remained for assessment following constraints and filtering. (A) Box-whisker plot of the pH values reported in the sample information (Mann-Whitney U statistic = 7,280, *P* < 9.95 × 10^−65^). (B and C) Regressions of the reported pH values against the first principal coordinate (PC1) from unweighted (B) and weighted (C) UniFrac analysis (Pearson *r* = 0.552, *P* < 6.61 × 10^−46^, and *r* = 0.562, *P* < 6.8 × 10^−48^, respectively). (D to G) Principal-coordinate plots of unweighted (D) and weighted (E) UniFrac of the observed samples colored by pH and unweighted (F) and weighted (G) UniFrac colored by the Qiita study identifier. (See Table S2 for additional study information.)

To test the search capability, we obtained sOTUs from a novel differential abundance method (12) in which five sOTUs were observed to strongly associate with high-pH soils and five with low-pH soils (see Table S1 in the supplemental material), in a reanalysis of a study by Ramirez et al. (13). We sought to determine whether the pH association of these sOTUs replicated across studies. Each sOTU was searched against 137,678 samples using redbiom, resulting in a total of 560 unique samples from 20 different Qiita studies (see the observed studies in Table S2 and the bash script for search in Text S1 in the supplemental material); a sample was only pulled out of Qiita if it contained any of the five high-pH or five low-pH sOTUs of interest. We did not calculate the prevalence of an sOTU because the interpretation may be misleading given inherent biases in which studies are represented in Qiita, different depths of sampling among different studies, etc. The search for samples, extraction of Deblur-processed data in BIOM format, and retrieval of sample metadata was performed per feature and took an average of 20 s. The pH of the observed samples was significantly different depending on the source feature set (Fig. 2A; Mann-Whitney U statistic = 7,280, *P* < 9.95 × 10^−65^). We then rarefied the samples to 1,000 sequences per sample and performed UniFrac (14) and principal-coordinate analysis on the collected samples, observing pH as a driver of community composition (Fig. 2B, unweighted Unifrac, Pearson’s *r* = 0.552, *P* < 6.61 × 10^−46^; Fig. 2C, weighted UniFrac, *r* = 0.562, *P* < 6.8 × 10^−48^). Visualization of the coordinates shows a visual pH gradient despite some study grouping (Fig. 2D to G), which is expected given the design of some studies (e.g., the *Cannabis* soil microbiome [15]). The analysis indicates that pH is a driver of overall community structure across multiple projects from a variety of institutions with markedly different research questions, soils, and locations.

10.1128/mSystems.00215-19.1 TEXT S1 A BASH script that performs the search in support of Fig. 2. Download Text S1, TXT file, 0.1 MB. Copyright © 2019 McDonald et al. 2019 McDonald et al. This content is distributed under the terms of the Creative Commons Attribution 4.0 International license.

10.1128/mSystems.00215-19.2 TABLE S1 The features searched for in the meta-analysis in Fig. 2. Download Table S1, XLSX file, 0.1 MB. Copyright © 2019 McDonald et al. 2019 McDonald et al. This content is distributed under the terms of the Creative Commons Attribution 4.0 International license.

10.1128/mSystems.00215-19.3 TABLE S2 Studies used in the meta-analysis presented in Fig. 2. Download Table S2, XLSX file, 0.1 MB. Copyright © 2019 McDonald et al. 2019 McDonald et al. This content is distributed under the terms of the Creative Commons Attribution 4.0 International license.

redbiom provides a critical part of the Earth Microbiome Project (5) infrastructure, underpinning the popular Trading Cards, with a default database that is regularly updated as new data are made public in Qiita (6). Additionally, redbiom allows queries across processing partitions, allowing users to operate across technical parameters if needed (e.g., to identify samples by Deblur and retrieve closed reference OTUs), as well as searching for samples by taxonomy when taxonomic information is present. These issues and others are explored in detail in a community tutorial for using redbiom with QIIME 2 (16), which together with the forum, the BSD open source license, and compatibility with microbiome standards will promote a broad user community. Finally, we note that the data model on which redbiom depends is general, allowing storage of gene expression and metabolomics data, and we expect that redbiom will provide a key underpinning for future multiomics microbiome studies as these capacities expand in the field.

## References

[1] Sinha R, Abu-Ali G, Vogtmann E, Fodor AA, Ren B, Amir A, Schwager E, Crabtree J, Ma S, Microbiome Quality Control Project Consortium, Abnet CC, Knight R, White O, Huttenhower C 2017 Assessment of variation in microbial community amplicon sequencing by the Microbiome Quality Control (MBQC) project consortium. Nat Biotechnol 35:1077–1086. doi:10.1038/nbt.3981. 28967885 PMC5839636

[2] Lozupone CA, Knight R 2007 Global patterns in bacterial diversity. Proc Natl Acad Sci U S A 104:11436–11440. doi:10.1073/pnas.0611525104. 17592124 PMC2040916

[3] Ley RE, Lozupone CA, Hamady M, Knight R, Gordon JI 2008 Worlds within worlds: evolution of the vertebrate gut microbiota. Nat Rev Microbiol 6:776–788. doi:10.1038/nrmicro1978. 18794915 PMC2664199

[4] Lozupone CA, Stombaugh J, Gonzalez A, Ackermann G, Wendel D, Vázquez-Baeza Y, Jansson JK, Gordon JI, Knight R 2013 Meta-analyses of studies of the human microbiota. Genome Res 23:1704–1714. doi:10.1101/gr.151803.112. 23861384 PMC3787266

[5] Thompson LR, Sanders JG, McDonald D, Amir A, Ladau J, Locey KJ, Prill RJ, Tripathi A, Gibbons SM, Ackermann G, Navas-Molina JA, Janssen S, Kopylova E, Vázquez-Baeza Y, González A, Morton JT, Mirarab S, Zech Xu Z, Jiang L, Haroon MF, Kanbar J, Zhu Q, Jin Song S, Kosciolek T, Bokulich NA, Lefler J, Brislawn CJ, Humphrey G, Owens SM, Hampton-Marcell J, Berg-Lyons D, McKenzie V, Fierer N, Fuhrman JA, Clauset A, Stevens RL, Shade A, Pollard KS, Goodwin KD, Jansson JK, Gilbert JA, Knight R, Earth Microbiome Project Consortium. 2017 A communal catalogue reveals Earth’s multiscale microbial diversity. Nature 551:457–463. doi:10.1038/nature24621. 29088705 PMC6192678

[6] Gonzalez A, Navas-Molina JA, Kosciolek T, McDonald D, Vázquez-Baeza Y, Ackermann G, DeReus J, Janssen S, Swafford AD, Orchanian SB, Sanders JG, Shorenstein J, Holste H, Petrus S, Robbins-Pianka A, Brislawn CJ, Wang M, Rideout JR, Bolyen E, Dillon M, Caporaso JG, Dorrestein PC, Knight R 2018 Qiita: rapid, web-enabled microbiome meta-analysis. Nat Methods 15:796–798. doi:10.1038/s41592-018-0141-9. 30275573 PMC6235622

[7] Yilmaz P, Kottmann R, Field D, Knight R, Cole JR, Amaral-Zettler L, Gilbert JA, Karsch-Mizrachi I, Johnston A, Cochrane G, Vaughan R, Hunter C, Park J, Morrison N, Rocca-Serra P, Sterk P, Arumugam M, Bailey M, Baumgartner L, Birren BW, Blaser MJ, Bonazzi V, Booth T, Bork P, Bushman FD, Buttigieg PL, Chain PSG, Charlson E, Costello EK, Huot-Creasy H, Dawyndt P, DeSantis T, Fierer N, Fuhrman JA, Gallery RE, Gevers D, Gibbs RA, San Gil I, Gonzalez A, Gordon JI, Guralnick R, Hankeln W, Highlander S, Hugenholtz P, Jansson J, Kau AL, Kelley ST, Kennedy J, Knights D, Koren O, 2011 Minimum information about a marker gene sequence (MIMARKS) and minimum information about any (x) sequence (MIxS) specifications. Nat Biotechnol 29:415–420. doi:10.1038/nbt.1823. 21552244 PMC3367316

[8] McDonald D, Clemente JC, Kuczynski J, Rideout JR, Stombaugh J, Wendel D, Wilke A, Huse S, Hufnagle J, Meyer F, Knight R, Caporaso JG 2012 The Biological Observation Matrix (BIOM) format or: how I learned to stop worrying and love the ome-ome. Gigascience 1:7. doi:10.1186/2047-217X-1-7. 23587224 PMC3626512

[9] Amir A, McDonald D, Navas-Molina JA, Kopylova E, Morton JT, Zech Xu Z, Kightley EP, Thompson LR, Hyde ER, Gonzalez A, Knight R 2017 Deblur rapidly resolves single-nucleotide community sequence patterns. mSystems 2:e00191-16. doi:10.1128/mSystems.00191-16. 28289731 PMC5340863

[10] Porter MF 1980 An algorithm for suffix stripping. Programmirovanie 14:130–137. doi:10.1108/eb046814.

[11] Callahan BJ, McMurdie PJ, Holmes SP 2017 Exact sequence variants should replace operational taxonomic units in marker-gene data analysis. ISME J 11:2639–2643. doi:10.1038/ismej.2017.119. 28731476 PMC5702726

[12] Morton J, Marotz C, Washburne A, Silverman J, Zaramela L, Edlund A, Zengler K, Knight R Establishing microbial composition measurement standards with reference frames. Nat Commun, in press. 10.1038/s41467-019-10656-5 PMC6586903 31222023

[13] Ramirez KS, Leff JW, Barberán A, Bates ST, Betley J, Crowther TW, Kelly EF, Oldfield EE, Shaw EA, Steenbock C, Bradford MA, Wall DH, Fierer N 2014 Biogeographic patterns in below-ground diversity in New York City’s Central Park are similar to those observed globally. Proc Biol Sci 281:20141988. doi:10.1098/rspb.2014.1988. 25274366 PMC4213626

[14] McDonald D, Vázquez-Baeza Y, Koslicki D, McClelland J, Reeve N, Xu Z, Gonzalez A, Knight R 2018 Striped UniFrac: enabling microbiome analysis at unprecedented scale. Nat Methods 15:847–848. doi:10.1038/s41592-018-0187-8. 30377368 PMC7250580

[15] Winston ME, Hampton-Marcell J, Zarraonaindia I, Owens SM, Moreau CS, Gilbert JA, Hartsel JA, Hartsel J, Kennedy SJ, Gibbons SM 2014 Understanding cultivar-specificity and soil determinants of the cannabis microbiome. PLoS One 9:e99641. doi:10.1371/journal.pone.0099641. 24932479 PMC4059704

[16] Bolyen E, Rideout JR, Dillon MR, Bokulich NA, Abnet C, Al-Ghalith GA, Alexander H, Alm EJ, Arumugam M, Asnicar F, Bai Y, Bisanz JE, Bittinger K, Brejnrod A, Brislawn CJ, Brown CT, Callahan BJ, Caraballo-Rodríguez AM, Chase J, Cope E, Da Silva R, Dorrestein PC, Douglas GM, Durall DM, Duvallet C, Edwardson CF, Ernst M, Estaki M, Fouquier J, Gauglitz JM, Gibson DL, Gonzalez A, Gorlick K, Guo J, Hillmann B, Holmes S, Holste H, Huttenhower C, Huttley G, Janssen S, Jarmusch AK, Jiang L, Kaehler B, Kang KB, Keefe CR, Keim P, Kelley ST, Knights D, Koester I, Kosciolek T, 2018 QIIME 2: reproducible, interactive, scalable, and extensible microbiome data science. PeerJ Preprints 6:e27295v2. doi:10.7287/peerj.preprints.27295v2. PMC7015180 31341288

